# Cost-Effectiveness of Endoscopic Bariatric Therapies Compared with Laparoscopic Sleeve Gastrectomy and Semaglutide

**DOI:** 10.1007/s11695-026-08854-4

**Published:** 2026-07-22

**Authors:** Nafsika Afentou, James Hall, Chidubem Okeke Ogwulu, Esther Albon, Janine Dretzke, Malcolm Price, Ken Clare, Rishi Singhal, Abd Tahrani, David J Moore, Emma Frew

**Affiliations:** 1 University of Birmingham, Birmingham, UK; 2 Canadian University of Dubai, Dubai, United Arab Emirates; 3 University Hospitals Birmingham NHS Foundation Trust, Birmingham, UK

## Abstract

**Introduction:**

Endoscopic bariatric therapies, including endoscopic sleeve gastroplasty (ESG) and intragastric balloon (IGB), are emerging as less invasive alternatives to bariatric surgery. However, their economic value relative to established treatments such as bariatric surgery and pharmacotherapy remains uncertain. This study evaluated the cost-effectiveness of endoscopic bariatric therapies compared with surgery and semaglutide from a UK health system perspective.

**Methods:**

Three state-transition Markov models compared: (1) ESG versus laparoscopic sleeve gastrectomy (LSG), (2) ESG versus semaglutide, and (3) IGB versus semaglutide. The models simulated transitions between body mass index (BMI)-defined health states over a 5-year time horizon using treatment effectiveness data derived from published studies and expert input. Outcomes were expressed as quality-adjusted life years (QALYs) and incremental cost-effectiveness ratios (ICERs) from the UK National Health Service perspective. Deterministic and probabilistic sensitivity analyses explored uncertainty.

**Results:**

For patients with class II-III obesity, LSG was cost-effective compared with ESG (£10,593/QALY). For class I and II obesity, ESG generated modestly greater QALYs than semaglutide at slightly higher cost (ICER £7,267/QALY). Semaglutide was dominant compared with IGB, producing greater health gains at lower cost. Results were most sensitive to intervention costs and assumptions regarding long-term recurrent weight gain.

**Conclusions:**

Endoscopic bariatric therapies may represent a cost-effective option for obesity management depending on patient characteristics and comparator treatments. LSG remains the most cost-effective intervention for severe obesity, whereas ESG may offer a cost-effective alternative to pharmacotherapy for patients with lower BMI.

**Supplementary Information:**

The online version contains supplementary material available at https://doi.org/10.1007/s11695-026-08854-4.

## Key Points

LSG was cost-effective compared with ESG for class II-III obesity.

ESG was cost-effective vs. semaglutide for class I-II obesity.

Semaglutide dominated intragastric balloon therapy.

Endoscopic therapies may bridge drugs and bariatric surgery.

**Supplementary Information:**

The online version contains supplementary material available at https://doi.org/10.1007/s11695-026-08854-4.

## Introduction

Obesity is associated with substantial morbidity, reduced quality-of-life, and increased health care utilization [1–4]. Bariatric surgery remains the most effective treatment for severe obesity, producing substantial and durable weight loss and improvements in obesity-related medical problems [5]. However, access to bariatric surgery remains limited in many health systems due to cost, health system capacity, and patient acceptability [6]. Pharmacologic therapies have expanded treatment options for patients with obesity. Glucagon-like peptide-1 receptor agonists such as semaglutide have demonstrated substantial short-term weight loss and are increasingly incorporated into clinical treatment pathways [7]. However, pharmacotherapy generally requires ongoing treatment and its long-term cost-effectiveness relative to procedural interventions remains uncertain [7]. Endoscopic bariatric therapies have emerged as less invasive alternatives to surgery. Procedures such as endoscopic sleeve gastroplasty (ESG) reduce gastric volume using endoscopic suturing techniques, while intragastric balloons (IGB) promote early satiety by occupying gastric space. These procedures may offer clinically meaningful weight loss with fewer complications than surgery and may be more acceptable to some patients [8–11].

Although clinical evidence supporting endoscopic bariatric therapies is increasing, their role within obesity treatment pathways remains uncertain. Limited evidence exists regarding their cost-effectiveness relative to established treatments such as bariatric surgery and pharmacotherapy. Economic evaluation is important for informing treatment pathways and healthcare resource allocation. The aim of this study was therefore to evaluate the cost-effectiveness of endoscopic bariatric therapies compared with bariatric surgery and pharmacotherapy using model-based economic evaluation from the perspective of the UK National Health Service (NHS).

## Methods

### Model Overview

Three state transition cohort Markov models were developed to evaluate the cost-effectiveness of endoscopic bariatric therapies relative to surgical and pharmacologic treatments. The models simulated patient transitions between BMI-defined health states reflecting changes in weight over time. Health states included no obesity, obesity class I, obesity class II, and severe obesity (class III). Each health state was associated with specific healthcare costs, quality-of-life estimates, and mortality risks. Three treatment comparisons were evaluated:

1. ESG versus laparoscopic sleeve gastrectomy (LSG)
2. ESG versus semaglutide
3. IGB versus semaglutide

**Fig. 1:**
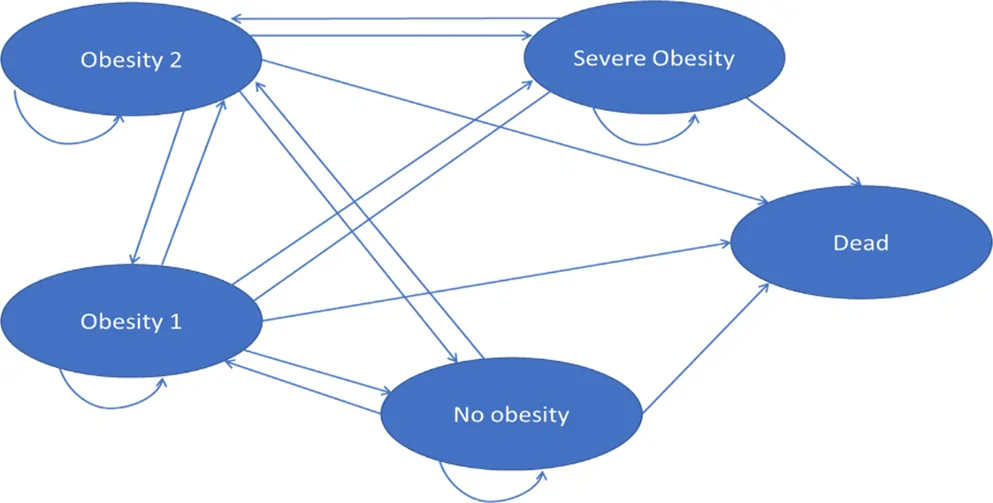
Model structure

Comparators were selected based on clinical practice, NICE guidance, and the NIHR-funded HTA stakeholder advisory group [12]. LSG was selected as the surgical comparator as it is the most performed bariatric procedure in UK practice and the primary surgical comparator in the available ESG evidence base [12]. Semaglutide was selected as it underpinned NICE guidance and NHS implementation at the time of model development [13]. The model cycle length was six months during the first year to capture early weight loss and one year thereafter. Half-cycle corrections were applied. The base-case time horizon was five years due to limitations in long-term treatment effectiveness data. Costs and quality-adjusted life years (QALYs) were evaluated from the perspective of the UK National Health Service and Personal Social Services and discounted at 3.5% annually, in accordance with National Institute for Health and Care Excellence (NICE) guidance [14]. Procedure-related mortality was modeled separately from BMI-related background mortality, which was incorporated using BMI-specific all-cause mortality hazard ratios. Additional model details are provided in the Supplementary Appendix A-C. The model structure is shown in Fig. 1.

### Baseline Population

Model populations were derived from patients eligible for obesity treatments according to UK NICE recommendations [7].

- Model 1: adults with obesity class II and III
- Models 2 and 3: adults with obesity class I and II

These eligibility groups reflect UK NICE guidance and routine NHS practice, where bariatric surgery is primarily performed in patients with class II-III obesity, while pharmacologic and less invasive interventions are more commonly used in patients with class I-II obesity. Baseline BMI distributions were used to allocate patients to health states at model entry. Patient characteristics were derived from a sample of 35,000 patients in the Clinical Practice Research Datalink (CPRD) database, accessed under appropriate governance approvals [15]. The CPRD dataset provides large-scale, real-world primary data for the UK and has been widely used in epidemiological and health economic research. This approach allowed the model to reflect the heterogeneity of patients eligible for obesity treatments in routine clinical practice. The mean age was 44 years and 87% were female. Average BMI was 40.3 (ranging from 35 to 87) for Model 1, and 33.52 (ranging from 30 to 39.99) for Models 2 and 3.

### Model Inputs

#### Treatment Effectiveness

Treatment effectiveness was parameterized using percentage total body weight loss derived from published studies identified through an evidence map and targeted literature searches [12] (see Table 1). Where direct comparative evidence was unavailable, indirect comparisons were conducted to estimate relative treatment effects. Weight loss trajectories were modeled over time and translated into transitions between BMI states. Where long-term data were unavailable, recurrent weight gain assumptions were informed by clinical input. All parameters along with their distributions and data sources are available in the Supplementary Appendix D Table S4.

**Table 1:** Effectiveness (% Total Body Weight Loss) for each treatment option

Model timepoint (months)	Treatment (% Total Body Weight Loss)			
	ESG	LSG	Semaglutide	IGB
6	15.2^a^	18.8^a^	13.05^e^	13.42^e^
12	19.1^a^	28.9^a^	18.56^e^	10.65^e^
24	16.4^a^	22.3^a^	19.06^f^	5.58^h^
36	14^b^	22.11^d^	12.71^g^	4.05^h^
48	11.9^c^	21.80^d^	6.35^g^	3.65^h^
60	9.97^c^	21.50^d^	0^g^	3.23^h^

^a^Beran, et al. [16]

^b^Rate of regain yr2-3 taken from Alqahtani et al. [17]

^c^Rate of change in regain year 1-3 assumed to continue in year 3-5

^d^Rate of regain year 2-5 taken from Wölnerhanssen, et al. [18]

^e^Indirect comparison with ESG

^f^Assumed another 0.5% weight loss in second year, as Wilding, et al. [19] showed increased weight loss from 52 weeks to 68 weeks

^g^Rapid recurrent weight gain after ceasing treatment based upon clinical assumption

^h^Kotzampassi, et al. [20]–Scaled their rate of recurrent weight gain yr1-5 to the weight lost at 12 months from indirect comparison

#### Utilities

Health state utilities were derived from a UK cost-effectiveness analysis of ESG and assigned according to BMI category [21]. Temporary utility decrements were applied to reflect treatment procedures and severe adverse events requiring hospitalization (see Supplementary Appendix D, Table S4). Utilities were based on BMI-defined health states rather than treatment-specific effects.

#### Costs

Intervention costs included procedural costs, follow-up care, and costs associated with obesity-related medical problems including type 2 diabetes, coronary heart disease, cardiovascular disease, sleep apnea, osteoarthritis – see supplementary Table S4. This approach allowed the model to capture the downstream economic impact of weight loss in obesity-related disease burden. These costs were applied according to BMI category (Table 2). All costs are reported in GBP prices for 2022/23.

**Table 2:** Costs associated with each model health state

Medical problems (prevalence, proportion)	Annual comorbidity cost (£)	Total cost (£)
Obesity III		**588.85**
T2DM (0.139)	318.28	
CHD (0.023)	42.03	
CVD (0.018)	73.70	
Sleep apnoea (0.018)	12.91	
Osteoarthritis (0.141)	141.94	
Obesity II		**506.10**
T2DM (0.099)	226.69	
CHD (0.032)	58.48	
CVD (0.02)	81.88	
Sleep apnoea (0.01)	7.17	
Osteoarthritis (0.13)	131.87	
Obesity I		**497.18**
T2DM (0.08)	183.19	
CHD (0.04)	73.10	
CVD (0.026)	106.45	
Sleep apnoea (0.005)	3.59	
Osteoarthritis (0.13)	130.86	
No obesity		**341.32**
T2DM (0.0383)	87.70	
CHD (0.0344)	62.92	
CVD (0.0235)	96.21	
Sleep apnoea (0.005)	3.59	
Osteoarthritis (0.0903)	90.90	

*T2DM *Type-2 diabetes mellitus, *CHD* Coronary heart disease, *CVD *Cardiovascular disease

*Annual management costs were estimated at £2290 for T2DM [22], £1828 for CHD (NHS cost 2021/22), £4094 for CVD (NHS cost 2021/22), £717 for Sleep Apnoea (NHS cost 2021/22), and £1007 for Osteoarthritis [23]

#### Outcomes and Analyses

The primary outcome was the incremental cost-effectiveness ratio (ICER) expressed as cost per QALY gained. Cost-effectiveness was assessed using the NICE willingness-to-pay (WTP) threshold (£20,000-£30,000 per QALY). Uncertainty was evaluated using both deterministic and probabilistic sensitivity analysis. Scenario analysis examined alternative assumptions regarding treatment duration for semaglutide. Model validation included internal consistency checks and external review against published evidence and clinical expectation; further details, including assessment of model-predicted health state occupancy, are provided in the Supplementary Appendix.

## Results

### Base-Case Analysis

Results of the base-case analysis are summarized in Table 3. For patients with class II-III obesity, LSG generated greater health gains than ESG but at higher cost. Over the 5-year time horizon, LSG produced 3.50 QALYs compared with 3.27 QALYs for ESG. The incremental cost-effectiveness ratio for LSG compared with ESG was £10,593 per QALY gained, indicating that LSG was cost-effective relative to ESG. For patients with class I-II obesity, ESG generated slightly greater health gains than semaglutide but at slightly higher cost. ESG produced 0.054 additional QALYs at an incremental cost of £394, resulting in an ICER of £7,267 per QALY gained. In contrast, semaglutide was dominant compared with IGB, producing slightly greater QALYs at lower cost.

**Table 3:** Summary of base-case results

Analysis	Cost (£)	QALY	ICER (£/QALY)
Model 1 (ESG vs. LSG)			
ESG	6425.10	3.2735	
LSG	8863.44	3.5037	
Difference*	-2438	-0.2302	(LSG) 10,593
Model 2 (ESG vs. semaglutide)			
ESG	6223.58	3.5420	
Semaglutide	5829.35	3.4878	
Difference*	394.23	0.0543	(ESG) 7,267
Model 3 (IGB vs. semaglutide)			
IGB	6432.16	3.4385	
Semaglutide	5829.09	3.4878	
Difference*	603.07	-0.0492	Semaglutide was dominant

*ICER *Incremental cost-effectiveness ratio, *ESG *Endoscopic sleeve gastroplasty, *LSG *Laparoscopic sleeve gastrectomy, *IGB *Intragastric balloon

*Incremental differences are presented as comparator minus reference intervention for each model (Model 1: ESG minus LSG; Models 2 and 3: ESG/IGB minus Semaglutide). Negative values therefore indicate lower costs or QALYs relative to the comparator

### Drivers of Cost-Effectiveness

Differences in cost-effectiveness were primarily driven by the magnitude and durability of weight loss achieved with LSG resulting in more patients transitioning to lower BMI health states over time. In the ESG versus semaglutide model, cost-effectiveness was influenced by assumptions regarding recurrent weight gain following discontinuation of pharmacotherapy. Under base-case assumptions, patients receiving semaglutide experienced gradual recurrent weight gain after treatment cessation, reducing long-term health gains relative to ESG. For the comparison between IGB and semaglutide, the relatively modest weight loss associated with IGB combined with procedural costs resulted in semaglutide being the dominant strategy.

### Sensitivity Analysis

Sensitivity analyses indicated that model results were most influenced by assumptions regarding treatment costs and long-term recurrent weight gain. In the ESG versus semaglutide model, reductions in ESG costs increased the probability that ESG would become the dominant treatment strategy. Conversely, more optimistic assumptions regarding long-term weight loss with semaglutide increased the ICER for ESG. In the scenario analysis extending semaglutide treatment to 5 years, ESG became dominant over semaglutide in Model 2, whereas in Model 3 semaglutide remained more effective than IGB but was no longer cost-effective (ICER £100,147/QALY). These findings were driven by increased cumulative treatment costs and ongoing treatment-related disutility associated with prolonged pharmacotherapy. This reflects the strong influence of structural assumptions, particularly those related to treatment duration, weight regain following discontinuation, and treatment-related disutility, on the ESG versus semaglutide comparison. Probabilistic sensitivity analysis demonstrated a high probability of cost-effectiveness for LSG compared with ESG. At a WTP threshold of £20,000 per QALY, LSG had a 99% probability of being cost-effective. ESG had an 85% probability of being cost-effective compared with semaglutide at the same threshold (see Supplementary Appendix E for more details).

## Discussion

This study evaluated the cost-effectiveness of endoscopic bariatric therapies compared with surgical and pharmacologic treatments using three economic models. The findings suggest that the relative value of these interventions varies according to both the severity of obesity and the comparator treatment. LSG was likely to be the most cost-effective intervention for patients with class II-III obesity, whereas ESG appeared cost-effective compared with semaglutide for patients with class I-II obesity. In contrast, semaglutide was dominant compared with IGB, producing slightly greater health gains at lower cost.

The greater cost-effectiveness of LSG compared with ESG was primarily driven by the magnitude and durability of weight loss associated with bariatric surgery. Although LSG involves higher upfront procedural costs, the larger and more sustained reductions in BMI resulted in greater long-term improvements in QALYs. These findings are consistent with the broader clinical literature demonstrating that bariatric surgery remains the most effective intervention for achieving durable weight loss in patients with severe obesity [5]. For patients with class I-II obesity, ESG was estimated to be cost-effective compared with semaglutide under the base-case assumptions. This comparison was particularly sensitive to assumptions regarding the duration of pharmacotherapy, with scenario analysis demonstrating that extending semaglutide treatment to 5 years could change the cost-effectiveness conclusions. The ESG versus semaglutide comparison is therefore driven to a large extent by these structural assumptions rather than robust long-term comparative evidence. While semaglutide has demonstrated substantial short-term weight loss in clinical trials, uncertainty remains regarding long-term weight trajectories once treatment is stopped. If pharmacotherapy were continued for longer durations, the relative cost-effectiveness of ESG may change. Scenario analysis extending semaglutide treatment suggested that increased drug costs could substantially influence the comparative economic value of pharmacotherapy and endoscopic procedures. Semaglutide was dominant compared with IGB in the modeled population. Although IGB can produce meaningful short-term weight loss, the magnitude and durability of weight reduction appear smaller than those observed with semaglutide. When combined with the procedural costs associated with balloon insertion and removal, these differences limited the cost-effectiveness of IGB within the modeled treatment pathway.

Overall, these findings suggest that endoscopic therapies may occupy an intermediate position within obesity treatment pathways between pharmacotherapy and bariatric surgery. Endoscopic procedures may be particularly relevant for patients who are not candidates for bariatric surgery or who prefer procedural interventions over long-term pharmacologic treatment. As the range of available obesity treatments continues to expand, understanding the relative clinical and economic value of these interventions will become increasingly important for informing treatment pathways and healthcare resource allocation.

Several limitations should be considered when interpreting the results of this study. First, limited long-term comparative evidence necessitated structural assumptions regarding weight regain. Second, treatment comparisons relied largely on indirect evidence due to the absence of randomized controlled trials directly comparing endoscopic therapies with pharmacologic treatments. Third, the analysis used a five-year time horizon due to limitations in available long-term comparative evidence across interventions. While longer-term outcomes are well established for bariatric surgery, equivalent data for endoscopic therapies and pharmacologic treatments remain limited. Extending the time horizon would therefore require additional assumptions regarding long-term weight trajectories, introducing greater uncertainty into the model.

The pharmacologic treatment landscape for obesity is evolving rapidly, and newer agents such as tirzepatide have demonstrated greater weight loss than semaglutide in recent clinical trials [24]. However, semaglutide was used as the comparator as it underpinned NHS implementation during model development.

In addition, health-related quality of life was modeled using BMI-based utility estimates rather than treatment-specific utilities, reflecting the availability of consistent data across interventions. Although procedure-related adverse events were included, long-term treatment-specific complications, such as gastroesophageal reflux disease and malnutrition, were not explicitly modeled. Furthermore, uncertainty remains regarding longer-term maintenance of weight loss beyond the available evidence base.

Finally, not all relevant comparators were included, and results should be interpreted in the context of comparator selection reflecting the available evidence base and NHS practice at the time of model development [24]. 

Future research should focus on generating longer-term comparative evidence for endoscopic bariatric therapies, particularly in relation to pharmacologic treatments such as semaglutide. Studies that reflect real-world treatment pathways, including sequential or combination therapies, would further improve understanding of the role of endoscopic interventions within comprehensive obesity management strategies.

## Conclusions

Endoscopic bariatric therapies may represent a cost-effective treatment option for obesity management depending on patient characteristic, comparator treatments, and assumptions regarding longer-term outcomes. LSG appeared to be the most cost-effective intervention for patients with severe obesity, whereas ESG may offer a cost-effective alternative to pharmacotherapy for patients with lower BMI. These findings are sensitive to assumptions regarding treatment duration, weight regain, and the evolving pharmacologic treatment landscape. Additional long-term comparator data are needed to better define the role of endoscopic therapies within obesity treatment pathways.

## Supplementary Information

Supplementary Material 1 (DOCX 1.31 MB)

## Data Availability

The data that support the findings of this study are available from the CPRD, but restrictions apply to their availability. Data were accessed under license for this study and are not publicly available. Aggregated data used in the analysis are presented within the article and supplementary materials.
