# Correction: Cost-Effectiveness of Endoscopic Bariatric Therapies Compared with Laparoscopic Sleeve Gastrectomy and Semaglutide

**DOI:** 10.1007/s11695-026-08885-x

**Published:** 2026-08-11

**Authors:** Nafsika Afentou, James Hall, Chidubem Okeke Ogwulu, Esther Albon, Janine Dretzke, Malcolm Price, Ken Clare, Rishi Singhal, Abd Tahrani, David J Moore, Emma Frew

**Affiliations:** 1 University of Birmingham, Birmingham, UK; 2 Canadian University of Dubai, Dubai, United Arab Emirates; 3 University Hospitals Birmingham NHS Foundation Trust, Birmingham, UK

**Correction: Obesity Surgery**

**https://doi.org/10.1007/s11695-026-08854-4**

The original article has been revised to correct typesetting errors in Table 1.

