# Supplementary material for: Cost-Effectiveness of Endoscopic Bariatric Therapies Compared with Laparoscopic Sleeve Gastrectomy and Semaglutide
Source: Obes Surg. 2026 Jul 22;36(9):4669–75. doi: 10.1007/s11695-026-08854-4 (PMC13562181; doi:10.1007/s11695-026-08854-4)
Supplement: Supplementary file 1 [file 11695_2026_8854_MOESM1_ESM.docx]

**Cost-effectiveness of Endoscopic Bariatric Therapies compared with Laparoscopic Sleeve Gastrectomy and Semaglutide**

**Supplementary Appendix**

This appendix provides additional detail on model structure, inputs, assumptions, and validation to support interpretation of the main analysis.

**Contents**

Appendix A. Model structure

Appendix B. Baseline population

Appendix C. Transition Probabilities

Appendix D. Model assumptions

Appendix E. Sensitivity analysis.

Appendix F. Model validation

**Appendix A. Model Structure**

This section describes the structure of the Markov models, including health states, transitions and comparators.

Three state-transition cohort Markov models were developed to estimate the cost-effectiveness of endoscopic bariatric therapies compared with surgical and pharmacologic treatments. Each model simulated transitions between BMI-defined health states over time.

The BMI health states included:

- No obesity
- Obesity class I
- Obesity class II
- Severe obesity (class III)

Transitions between health states were driven by treatment-specific weight loss and subsequent weight regain. Background mortality risk was applied according to BMI category using BMI-specific all-cause mortality hazard ratios.

The models compared the following interventions:

- **Model 1:** Endoscopic sleeve gastroplasty (ESG) versus laparoscopic sleeve gastrectomy (LSG)
- **Model 2:** ESG versus semaglutide
- **Model 3:** Intragastric balloon (IGB) versus semaglutide

The model cycle length was six months during the first year to capture early treatment effects and one year thereafter. Half-cycle corrections were applied. The base-case time horizon was five years, reflecting the availability of treatment effectiveness data. All models were implemented in Microsoft Excel 2018.

**Appendix B: Baseline population**

This section describes the baseline characteristics and BMI distribution of the simulated population.

To generate the starting positions of the model population, patients were allocated to baseline health states based on the BMI distribution derived from the UK Clinical Practice Research Datalink (CPRD) database, accessed under appropriate governance approvals[1]. A sample of approximately 35,000 patients with recorded BMI values was extracted. Eligibility criteria were applied to reflect patients who would typically qualify for the modeled treatments according to NICE guidance[2,3]. For Model 1 (ESG vs LSG), patients with **class II–III obesity (BMI ≥35 kg/m²)** were included. For Models 2 and 3 (endoscopic treatments vs semaglutide), patients with **class I–II obesity (BMI 30–39.9 kg/m²)** were included. The mean age of the population was 44 years and approximately 87% were female. Average BMI was 40.3 (ranging from 35 to 87) for Model 1, and 33.52 (ranging from 30 to 39.99) for Models 2 and 3.

Abbreviations: ESG = endoscopic sleeve gastroplasty; LSG = laparoscopic sleeve gastrectomy; IGB = intragastric balloon.

**Appendix C: Derivation of transition probabilities**

This section describes how transition probabilities between BMI-defined health states were derived from treatment-specific weight loss data. Transitions reflect movement between BMI-defined health states across model cycles.

Transition probabilities between BMI health states were derived from treatment-specific weight loss estimates. Effectiveness data were expressed as **percentage total body weight loss (%TBWL)** obtained from published clinical studies and indirect comparisons[4].

To translate weight loss estimates into BMI transitions:

1. Baseline patient weights from the CPRD dataset were updated for each cycle using the expected percentage weight change.
2. Individual-level weight trajectories were generated using Monte Carlo simulation to reflect variation in treatment response.
3. Updated BMI values were used to determine the corresponding BMI health state at each time point.
4. The proportion of patients transitioning between BMI states across model cycles was calculated and converted into transition probabilities.

Separate simulations were conducted for each intervention to capture uncertainty in treatment response.

The table below presents transition probabilities and corresponding alpha and beta parameters used in the probabilistic sensitivity analysis for Model 1 (ESG vs LSG). Transitions are presented as movements between BMI health states (e.g. ‘2>1 indicates a transition from obesity class II to class I).

**Table S1: Model 1 transition probabilities and alpha/beta values for probabilistic sensitivity analysis (PSA)**

| ESG | | | | LSG | | |
| --- | --- | --- | --- | --- | --- | --- |
| Transition | Alpha | Beta | Transition probability | Alpha | Beta | Transition probability |
| **Baseline>6m** | | | | | | |
| 0>0 |  |  |  |  |  |  |
| 0>1 |  |  |  |  |  |  |
| 0>2 |  |  |  |  |  |  |
| 0>3 |  |  |  |  |  |  |
| 1>0 |  |  |  |  |  |  |
| 1>1 |  |  |  |  |  |  |
| 1>2 |  |  |  |  |  |  |
| 1>3 |  |  |  |  |  |  |
| 2>0 | 244.79 | 565.21 | 0.30 | 370.80 | 369.20 | 0.50 |
| 2>1 | 487.84 | 322.16 | 0.60 | 323.03 | 416.97 | 0.44 |
| 2>2 | 75.78 | 734.22 | 0.09 | 44.44 | 695.56 | 0.06 |
| 2>3 | 1.59 | 808.41 | 0.00 | 1.74 | 738.26 | 0.00 |
| 3>0 | 2.05 | 271.95 | 0.01 | 15.42 | 234.58 | 0.06 |
| 3>1 | 68.08 | 205.92 | 0.25 | 87.58 | 162.42 | 0.35 |
| 3>2 | 114.34 | 159.66 | 0.42 | 83.13 | 166.87 | 0.33 |
| 3>3 | 89.52 | 184.48 | 0.33 | 63.88 | 186.12 | 0.26 |
| **6m>12m** | | | | | | |
| 0>0 | 246.85 | 0.00 | 1.00 | 386.22 | 0 | 1 |
| 0>1 | 0.00 | 246.85 | 0.00 | 0 | 386.22 | 0 |
| 0>2 | 0.00 | 246.85 | 0.00 | 0 | 386.22 | 0 |
| 0>3 | 0.00 | 246.85 | 0.00 | 0 | 386.22 | 0 |
| 1>0 | 186.27 | 369.66 | 0.34 | 347.71 | 62.88 | 0.85 |
| 1>1 | 369.59 | 186.34 | 0.66 | 62.89 | 347.71 | 0.15 |
| 1>2 | 0.07 | 555.85 | 0.00 | 0 | 410.60 | 0 |
| 1>3 | 0.00 | 555.92 | 0.00 | 0 | 410.60 | 0 |
| 2>0 | 0.00 | 190.12 | 0.00 | 0.60 | 126.96 | 0.01 |
| 2>1 | 68.51 | 121.61 | 0.36 | 115.28 | 12.28 | 0.90 |
| 2>2 | 121.09 | 69.03 | 0.64 | 11.68 | 115.89 | 0.09 |
| 2>3 | 0.52 | 189.60 | 0.00 | 0 | 127.56 | 0 |
| 3>0 | 0.00 | 91.11 | 0.00 | 0 | 65.62 | 0 |
| 3>1 | 0.00 | 91.11 | 0.00 | 0.99 | 64.63 | 0.02 |
| 3>2 | 20.66 | 70.45 | 0.23 | 34.07 | 31.54 | 0.52 |
| 3>3 | 70.45 | 20.66 | 0.77 | 30.55 | 35.06 | 0.47 |
| **12m>24m** | | | | | | |
| 0>0 | 354.22 | 78.89 | 0.82 | 503.40 | 231.1345 | 0.69 |
| 0>1 | 78.89 | 354.22 | 0.18 | 231.13 | 503.4048 | 0.31 |
| 0>2 | 0.00 | 433.11 | 0.00 | 0 | 734.5393 | 0 |
| 0>3 | 0.00 | 433.11 | 0.00 | 0 | 734.5393 | 0 |
| 1>0 | 1.36 | 436.74 | 0.00 | 0 | 179.1558 | 0 |
| 1>1 | 339.84 | 98.25 | 0.78 | 103.06 | 76.09931 | 0.56 |
| 1>2 | 96.89 | 341.20 | 0.22 | 76.10 | 103.0564 | 0.42 |
| 1>3 | 0.00 | 438.10 | 0.00 | 0 | 179.16 | 0 |
| 2>0 | 0.00 | 141.82 | 0.00 | 0 | 45.75 | 0 |
| 2>1 | 0.20 | 141.62 | 0.00 | 0 | 45.75 | 0 |
| 2>2 | 105.73 | 36.09 | 0.75 | 24.13 | 21.62 | 0.53 |
| 2>3 | 35.89 | 105.93 | 0.25 | 21.62 | 24.13 | 0.47 |
| 3>0 | 0.00 | 70.97 | 0.00 | 0 | 30.55 | 0 |
| 3>1 | 0.00 | 70.97 | 0.00 | 0 | 30.55 | 0 |
| 3>2 | 0.09 | 70.88 | 0.00 | 0 | 30.55 | 0 |
| 3>3 | 70.88 | 0.09 | 1.00 | 30.55 | 0 | 1 |
| **24m>36m** | | | | | | |
| 0>0 | 264.53 | 37.36 | 0.88 | 365.72 | 74.60 | 0.83 |
| 0>1 | 37.36 | 264.53 | 0.12 | 74.32 | 366.01 | 0.17 |
| 0>2 | 0.00 | 301.89 | 0.00 | 0.29 | 440.04 | 0.00 |
| 0>3 | 0.00 | 301.89 | 0.00 | 0 | 440.33 | 0 |
| 1>0 | 0.95 | 318.03 | 0.00 | 72.37 | 187.06 | 0.28 |
| 1>1 | 257.79 | 61.19 | 0.81 | 169.96 | 89.47 | 0.66 |
| 1>2 | 60.24 | 258.74 | 0.19 | 16.96 | 242.47 | 0.07 |
| 1>3 | 0.00 | 318.98 | 0.00 | 0.14 | 259.29 | 0.00 |
| 2>0 | 0.00 | 318.98 | 0.00 | 0.78 | 258.65 | 0.00 |
| 2>1 | 0.10 | 112.54 | 0.00 | 22.52 | 25.17 | 0.47 |
| 2>2 | 85.86 | 26.77 | 0.76 | 21.94 | 25.74 | 0.46 |
| 2>3 | 26.67 | 85.96 | 0.24 | 2.45 | 45.23 | 0.05 |
| 3>0 | 0.00 | 23.50 | 0.00 | 0 | 9.56 | 0 |
| 3>1 | 0.00 | 23.50 | 0.00 | 0.43 | 9.13 | 0.05 |
| 3>2 | 0.01 | 23.49 | 0.00 | 2.60 | 6.96 | 0.27 |
| 3>3 | 23.49 | 0.01 | 1.00 | 6.53 | 3.03 | 0.68 |
| **36m>48m** | | | | | | |
| 0>0 | 125.36 | 7.38 | 0.94 | 212.04 | 7.39 | 0.97 |
| 0>1 | 7.38 | 125.36 | 0.06 | 7.39 | 212.04 | 0.03 |
| 0>2 | 0.00 | 132.74 | 0.00 | 0 | 219.43 | 0 |
| 0>3 | 0.00 | 132.74 | 0.00 | 0 | 219.43 | 0 |
| 1>0 | 2.51 | 145.11 | 0.02 | 0.51 | 133.10 | 0.00 |
| 1>1 | 120.45 | 27.17 | 0.82 | 131.49 | 2.12 | 0.98 |
| 1>2 | 24.67 | 122.95 | 0.17 | 1.61 | 132.00 | 0.01 |
| 1>3 | 0.00 | 147.62 | 0.00 | 0 | 133.61 | 0 |
| 2>0 | 0.00 | 73.06 | 0.00 | 0 | 20.89 | 0 |
| 2>1 | 0.33 | 72.73 | 0.00 | 0.10 | 20.79 | 0.00 |
| 2>2 | 55.08 | 17.98 | 0.75 | 20.47 | 0.42 | 0.98 |
| 2>3 | 17.66 | 55.40 | 0.24 | 0.32 | 20.57 | 0.02 |
| 3>0 | 0.00 | 25.08 | 0.00 | 0 | 4.56 | 0 |
| 3>1 | 0.00 | 25.08 | 0.00 | 0 | 4.56 | 0 |
| 3>2 | 0.06 | 25.02 | 0.00 | 0.01 | 4.55 | 0.00 |
| 3>3 | 25.02 | 0.06 | 1.00 | 4.55 | 0.00 | 1.00 |
| **48m > 60m** | | | | | | |
| 0>0 | 105.03 | 22.85 | 0.82 | 205.83 | 6.73 | 0.97 |
| 0>1 | 22.85 | 105.03 | 0.18 | 6.73 | 205.83 | 0.03 |
| 0>2 | 0.00 | 127.87 | 0.00 | 0 | 212.56 | 0 |
| 0>3 | 0.00 | 127.87 | 0.00 | 0 | 212.56 | 0 |
| 1>0 | 0.00 | 128.15 | 0.00 | 0 | 138.98 | 0 |
| 1>1 | 106.41 | 21.74 | 0.83 | 137.02 | 1.96 | 0.99 |
| 1>2 | 21.74 | 106.41 | 0.17 | 1.96 | 137.02 | 0.01 |
| 1>3 | 0.00 | 128.15 | 0.00 | 0 | 138.98 | 0 |
| 2>0 | 0.00 | 79.81 | 0.00 | 0 | 22.09 | 0 |
| 2>1 | 0.00 | 79.81 | 0.00 | 0 | 22.09 | 0 |
| 2>2 | 68.16 | 11.65 | 0.85 | 21.73 | 0.36 | 0.98 |
| 2>3 | 11.65 | 68.16 | 0.15 | 0.36 | 21.73 | 0.02 |
| 3>0 | 0.00 | 42.67 | 0.00 | 0 | 4.87 | 0 |
| 3>1 | 0.00 | 42.67 | 0.00 | 0 | 4.87 | 0 |
| 3>2 | 0.00 | 42.67 | 0.00 | 0 | 4.87 | 0 |
| 3>3 | 42.67 | 0.00 | 1.00 | 4.87 | 0 | 1 |

The table below presents transition probabilities and corresponding alpha and beta parameters used in the probabilistic sensitivity analysis for Model 2 (ESG vs Semaglutide).

**Table S2: Model 2 transition probabilities and alpha/beta values for PSA**

|  | **ESG** | | | **Semaglutide (Also for model 3)** | | |
| --- | --- | --- | --- | --- | --- | --- |
| Transition | Alpha | Beta | Transition probability | Alpha | Beta | Transition probability |
| **Baseline>6m** | | | | | | |
| 0>0 |  |  |  |  |  |  |
| 0>1 |  |  |  |  |  |  |
| 0>2 |  |  |  |  |  |  |
| 0>3 |  |  |  |  |  |  |
| 1>0 | 1187.81 | 164.19 | 0.88 | 391.07 | 44.93 | 0.90 |
| 1>1 | 161.63 | 1190.37 | 0.12 | 44.93 | 391.07 | 0.10 |
| 1>2 | 2.51 | 1349.49 | 0.00 | 0 | 436 | 0 |
| 1>3 | 0.05 | 1351.95 | 0.00 | 0 | 436 | 0 |
| 2>0 | 288.98 | 670.02 | 0.30 | 32.69 | 373.31 | 0.08 |
| 2>1 | 582.28 | 376.72 | 0.61 | 351.15 | 54.85 | 0.86 |
| 2>2 | 86.90 | 872.10 | 0.09 | 22.15 | 383.85 | 0.05 |
| 2>3 | 0.85 | 958.15 | 0.00 | 0 | 406 | 0 |
| 3>0 |  |  |  |  |  |  |
| 3>1 |  |  |  |  |  |  |
| 3>2 |  |  |  |  |  |  |
| 3>3 |  |  |  |  |  |  |
| **6m>12m** | | | | | | |
| 0>0 | 1476.73 | 0.06 | 1.00 | 423.77 | 0 | 1 |
| 0>1 | 0 | 1476.79 | 0 | 0 | 423.77 | 0 |
| 0>2 | 0 | 1476.79 | 0 | 0 | 423.77 | 0 |
| 0>3 | 0 | 1476.79 | 0 | 0 | 423.77 | 0 |
| 1>0 | 264.46 | 479.45 | 0.36 | 221.25 | 174.83 | 0.56 |
| 1>1 | 477.92 | 265.98 | 0.64 | 174.83 | 221.24 | 0.44 |
| 1>2 | 1.52 | 742.39 | 0.00 | 0 | 396.08 | 0 |
| 1>3 | 0 | 743.91 | 0 | 0 | 396.08 | 0 |
| 2>0 | 0 | 89.41 | 0 | 0 | 22.15 | 0 |
| 2>1 | 27.93 | 61.47 | 0.31 | 21.12 | 1.03 | 0.95 |
| 2>2 | 60.65 | 28.76 | 0.68 | 1.03 | 21.12 | 0.05 |
| 2>3 | 0.83 | 88.58 | 0.01 | 0 | 22.15 | 0 |
| 3>0 | 0 | 0.90 | 0 | 0 | 0 | 0 |
| 3>1 | 0 | 0.90 | 0 | 0 | 0 | 0 |
| 3>2 | 0 | 0.90 | 0 | 0 | 0 | 0 |
| 3>3 | 0 | 0.90 | 0 | 0 | 0 | 0 |
| **12m>24m** | | | | | | |
| 0>0 | 1534.28 | 206.91 | 0.88 | 322.51 | 0 | 1 |
| 0>1 | 206.91 | 1534.28 | 0.12 | 0 | 322.51 | 0 |
| 0>2 | 0 | 1741.19 | 0 | 0 | 322.51 | 0 |
| 0>3 | 0 | 1741.19 | 0 | 0 | 322.51 | 0 |
| 1>0 | 0 | 505.86 | 0 | 8.48 | 89.50 | 0.09 |
| 1>1 | 405.72 | 100.14 | 0.80 | 89.50 | 8.48 | 0.91 |
| 1>2 | 100.14 | 405.72 | 0.20 | 0 | 97.98 | 0 |
| 1>3 | 0 | 505.86 | 0 | 0 | 97.98 | 0 |
| 2>0 | 0 | 62.17 | 0 | 0 | 0.52 | 0 |
| 2>1 | 0 | 62.17 | 0 | 0.24 | 0.28 | 0.46 |
| 2>2 | 42.67 | 19.50 | 0.69 | 0.28 | 0.24 | 0.54 |
| 2>3 | 19.50 | 42.67 | 0.31 | 0 | 0.52 | 0 |
| 3>0 | 0 | 0.83 | 0 | 0 | 0 |  |
| 3>1 | 0 | 0.83 | 0 | 0 | 0 |  |
| 3>2 | 0 | 0.83 | 0 | 0 | 0 |  |
| 3>3 | 0 | 0.83 | 0 | 0 | 0 |  |
| **24m>36m** | | | | | | |
| 0>0 | 1492.64 | 189.47 | 0.89 | 115.75 | 49.74 | 0.70 |
| 0>1 | 189.47 | 1492.64 | 0.11 | 46.01 | 119.48 | 0.28 |
| 0>2 | 0.00 | 1682.11 | 0.00 | 3.65 | 161.84 | 0.02 |
| 0>3 | 0.00 | 1682.11 | 0.00 | 0.07 | 165.42 | 0.00 |
| 1>0 | 0.00 | 539.52 | 0.00 | 1.31 | 43.56 | 0.03 |
| 1>1 | 432.78 | 106.75 | 0.80 | 21.57 | 23.30 | 0.48 |
| 1>2 | 106.75 | 432.78 | 0.20 | 18.79 | 26.08 | 0.42 |
| 1>3 | 0.00 | 539.52 | 0.00 | 3.20 | 41.67 | 0.07 |
| 2>0 | 0.00 | 539.52 | 0.00 | 0.00 | 44.87 | 0.00 |
| 2>1 | 0.00 | 105.30 | 0.00 | 0.00 | 0.14 | 0.00 |
| 2>2 | 81.62 | 23.67 | 0.78 | 0.00 | 0.14 | 0.00 |
| 2>3 | 23.67 | 81.62 | 0.22 | 0.14 | 0.00 | 1.00 |
| 3>0 | 0.00 | 12.85 | 0.00 | 0.00 | 0.00 | 0.00 |
| 3>1 | 0.00 | 12.85 | 0.00 | 0.00 | 0.00 | 0.00 |
| 3>2 | 0.00 | 12.85 | 0.00 | 0.00 | 0.00 | 0.00 |
| 3>3 | 12.85 | 0.00 | 1.00 | 0.00 | 0.00 | 1.00 |
| **36m>48m** | | | | | | |
| 0>0 | 673.76 | 72.57 | 0.90 | 38.72 | 19.81 | 0.66 |
| 0>1 | 72.57 | 673.76 | 0.10 | 19.81 | 38.72 | 0.34 |
| 0>2 | 0.00 | 746.32 | 0.00 | 0.00 | 58.53 | 0.00 |
| 0>3 | 0.00 | 746.32 | 0.00 | 0.00 | 58.53 | 0.00 |
| 1>0 | 0.19 | 310.93 | 0.00 | 0.00 | 33.79 | 0.00 |
| 1>1 | 246.27 | 64.85 | 0.79 | 22.78 | 11.01 | 0.67 |
| 1>2 | 64.66 | 246.46 | 0.21 | 11.01 | 22.78 | 0.33 |
| 1>3 | 0.00 | 311.12 | 0.00 | 0.00 | 33.79 | 0.00 |
| 2>0 | 0.00 | 94.19 | 0.00 | 0.00 | 11.22 | 0.00 |
| 2>1 | 0.00 | 94.19 | 0.00 | 0.00 | 11.22 | 0.00 |
| 2>2 | 66.75 | 27.43 | 0.71 | 8.29 | 2.93 | 0.74 |
| 2>3 | 27.43 | 66.75 | 0.29 | 2.93 | 8.29 | 0.26 |
| 3>0 | 0.00 | 18.26 | 0.00 | 0.00 | 1.71 | 0.00 |
| 3>1 | 0.00 | 18.26 | 0.00 | 0.00 | 1.71 | 0.00 |
| 3>2 | 0.00 | 18.26 | 0.00 | 0.00 | 1.71 | 0.00 |
| 3>3 | 18.26 | 0.00 | 1.00 | 1.71 | 0.00 | 1.00 |
| **48m > 60m** | | | | | | |
| 0>0 | 613.35 | 60.60 | 0.91 | 10.33 | 9.03 | 0.53 |
| 0>1 | 60.60 | 613.35 | 0.09 | 9.03 | 10.33 | 0.47 |
| 0>2 | 0.00 | 673.95 | 0.00 | 0.00 | 19.36 | 0.00 |
| 0>3 | 0.00 | 673.95 | 0.00 | 0.00 | 19.36 | 0.00 |
| 1>0 | 0.00 | 318.83 | 0.00 | 0.00 | 21.30 | 0.00 |
| 1>1 | 282.83 | 36.01 | 0.89 | 13.56 | 7.74 | 0.64 |
| 1>2 | 36.01 | 282.83 | 0.11 | 7.74 | 13.56 | 0.36 |
| 1>3 | 0.00 | 318.83 | 0.00 | 0.00 | 21.30 | 0.00 |
| 2>0 | 0.00 | 131.41 | 0.00 | 0.00 | 19.80 | 0.00 |
| 2>1 | 0.00 | 131.41 | 0.00 | 0.00 | 19.80 | 0.00 |
| 2>2 | 118.89 | 12.52 | 0.90 | 13.99 | 5.80 | 0.71 |
| 2>3 | 12.52 | 118.89 | 0.10 | 5.80 | 13.99 | 0.29 |
| 3>0 | 0.00 | 45.70 | 0.00 | 0.00 | 2.32 | 0.00 |
| 3>1 | 0.00 | 45.70 | 0.00 | 0.00 | 2.32 | 0.00 |
| 3>2 | 0.00 | 45.70 | 0.00 | 0.00 | 2.32 | 0.00 |
| 3>3 | 45.70 | 0.00 | 1.00 | 2.32 | 0.00 | 1.00 |

The table below presents transition probabilities and corresponding alpha and beta parameters used in the probabilistic sensitivity analysis for Model 3 (IGB vs Semaglutide).

**Table S3: Model 3 transition probabilities and alpha/beta values for PSA**

| **IGB** | | | |
| --- | --- | --- | --- |
| Transition | Alpha | Beta | Transition probability |
| **baseline>6m** | | | |
| 0>0 | 0.00 | 0.00 |  |
| 0>1 | 0.00 | 0.00 |  |
| 0>2 | 0.00 | 0.00 |  |
| 0>3 | 0.00 | 0.00 |  |
| 1>0 | 105.00 | 17.00 | 0.86 |
| 1>1 | 16.94 | 105.06 | 0.14 |
| 1>2 | 0.06 | 121.94 | 0.00 |
| 1>3 | 0.00 | 122.00 | 0.00 |
| 2>0 | 15.12 | 70.88 | 0.18 |
| 2>1 | 62.16 | 23.84 | 0.72 |
| 2>2 | 8.69 | 77.31 | 0.10 |
| 2>3 | 0.03 | 85.97 | 0.00 |
| 3>0 | 0.00 | 0.00 |  |
| 3>1 | 0.00 | 0.00 |  |
| 3>2 | 0.00 | 0.00 |  |
| 3>3 | 0.00 | 0.00 |  |
| **6m>12m** | | | |
| 0>0 | 42.50 | 8.18 | 0.84 |
| 0>1 | 0.00 | 50.68 | 0.00 |
| 0>2 | 0.00 | 50.68 | 0.00 |
| 0>3 | 0.00 | 50.68 | 0.00 |
| 1>0 | 0.00 | 33.37 | 0.00 |
| 1>1 | 30.21 | 3.16 | 0.91 |
| 1>2 | 3.16 | 30.21 | 0.09 |
| 1>3 | 0.00 | 33.37 | 0.00 |
| 2>0 | 0.00 | 3.69 | 0.00 |
| 2>1 | 0.00 | 3.69 | 0.00 |
| 2>2 | 3.65 | 0.04 | 0.99 |
| 2>3 | 0.04 | 3.65 | 0.01 |
| 3>0 | 0.00 | 0.01 | 0.00 |
| 3>1 | 0.00 | 0.01 | 0.00 |
| 3>2 | 0.00 | 0.01 | 0.00 |
| 3>3 | 0.01 | 0.00 | 1.00 |
| **12m>24m** | | | |
| 0>0 | 14.65 | 6.60 | 0.69 |
| 0>1 | 6.11 | 15.14 | 0.29 |
| 0>2 | 0.49 | 20.76 | 0.02 |
| 0>3 | 0.00 | 21.25 | 0.00 |
| 1>0 | 1.41 | 13.70 | 0.09 |
| 1>1 | 6.64 | 8.46 | 0.44 |
| 1>2 | 5.63 | 9.48 | 0.37 |
| 1>3 | 1.43 | 13.68 | 0.09 |
| 2>0 | 0.00 | 3.41 | 0.00 |
| 2>1 | 0.00 | 3.41 | 0.00 |
| 2>2 | 0.89 | 2.52 | 0.26 |
| 2>3 | 2.52 | 0.89 | 0.74 |
| 3>0 | 0.00 | 0.06 | 0.00 |
| 3>1 | 0.00 | 0.06 | 0.00 |
| 3>2 | 0.00 | 0.06 | 0.00 |
| 3>3 | 0.06 | 0.00 | 1.00 |
| **24m>36m** | | | |
| 0>0 | 14.66 | 1.39 | 0.91 |
| 0>1 | 1.39 | 14.66 | 0.09 |
| 0>2 | 0.00 | 16.06 | 0.00 |
| 0>3 | 0.00 | 16.06 | 0.00 |
| 1>0 | 0.00 | 12.75 | 0.00 |
| 1>1 | 11.66 | 1.10 | 0.91 |
| 1>2 | 1.10 | 11.66 | 0.09 |
| 1>3 | 0.00 | 12.75 | 0.00 |
| 2>0 | 0.00 | 12.75 | 0.00 |
| 2>1 | 0.00 | 7.00 | 0.00 |
| 2>2 | 6.52 | 0.48 | 0.93 |
| 2>3 | 0.48 | 6.52 | 0.07 |
| 3>0 | 0.00 | 4.01 | 0.00 |
| 3>1 | 0.00 | 4.01 | 0.00 |
| 3>2 | 0.00 | 4.01 | 0.00 |
| 3>3 | 4.01 | 0.00 | 1.00 |
| **36m>48m** | | | |
| 0>0 | 14.31 | 0.36 | 0.98 |
| 0>1 | 0.36 | 14.31 | 0.02 |
| 0>2 | 0.00 | 14.66 | 0.00 |
| 0>3 | 0.00 | 14.66 | 0.00 |
| 1>0 | 0.00 | 13.05 | 0.00 |
| 1>1 | 12.80 | 0.25 | 0.98 |
| 1>2 | 0.25 | 12.80 | 0.02 |
| 1>3 | 0.00 | 13.05 | 0.00 |
| 2>0 | 0.00 | 7.61 | 0.00 |
| 2>1 | 0.00 | 7.61 | 0.00 |
| 2>2 | 7.48 | 0.13 | 0.98 |
| 2>3 | 0.13 | 7.48 | 0.02 |
| 3>0 | 0.00 | 4.49 | 0.00 |
| 3>1 | 0.00 | 4.49 | 0.00 |
| 3>2 | 0.00 | 4.49 | 0.00 |
| 3>3 | 4.49 | 0.00 | 1.00 |
| **48m > 60m** | | | |
| 0>0 | 13.94 | 0.37 | 0.97 |
| 0>1 | 0.37 | 13.94 | 0.03 |
| 0>2 | 0.00 | 14.31 | 0.00 |
| 0>3 | 0.00 | 14.31 | 0.00 |
| 1>0 | 0.00 | 13.15 | 0.00 |
| 1>1 | 12.85 | 0.31 | 0.98 |
| 1>2 | 0.31 | 12.85 | 0.02 |
| 1>3 | 0.00 | 13.15 | 0.00 |
| 2>0 | 0.00 | 7.73 | 0.00 |
| 2>1 | 0.00 | 7.73 | 0.00 |
| 2>2 | 7.59 | 0.14 | 0.98 |
| 2>3 | 0.14 | 7.59 | 0.02 |
| 3>0 | 0.00 | 4.62 | 0.00 |
| 3>1 | 0.00 | 4.62 | 0.00 |
| 3>2 | 0.00 | 4.62 | 0.00 |
| 3>3 | 4.62 | 0.00 | 1.00 |

**Appendix D: Model assumptions**

This section outlines the key structural and parameter assumptions used in the model. Several modeling assumptions were required due to limitations in the available evidence.

Adverse events:

- Due to scarcity and inconsistencies in the existing data, coupled with the minimal sensitivity of the model to severe adverse events (SAEs), an aggregate approach was followed to model SAEs. Using this approach, estimates for incident rates, and utilities, related to all SAEs were averaged and incorporated into the model.
- SAEs related to surgical procedures included in the model were assumed to be intervention-related one-off events likely to occur once following the intervention, hence the costs and disutility relating to the SAEs were applied in the first model cycle (the first six months) only.

Semaglutide treatment assumptions:

- However, for semaglutide (which involves continuous treatment for two years), it was assumed that any SAEs could occur more than once, therefore, the relevant disutility and associated costs were applied per cycle up to two years after which treatment was withdrawn (as per NICE guidelines).[18]

Weight regain assumptions:

- For Model 1 and 2 (ESG versus LSG and ESG versus semaglutide) it was assumed that rates of weight regain for patients who had ESG occurred at the same rate for years four and five as in year two and three.
- For Model 2 (ESG versus semaglutide) mean %TBWL differences were obtained for ESG compared to semaglutide at six months and 12 months, from an indirect comparison of studies conducted by the team.
- Beyond 12 months, based on existing literature and clinical expert validation, it was assumed that following semaglutide treatment, patients maintained their weight loss until the end of the second year. Following expert advice, it was then assumed that all weight was regained in a linear fashion from the end of year two until year five.

Utilities:

- Health state utilities were assumed to be dependent on BMI. Consequently, it was assumed that utilities remained constant for each health state over time, aside from age-adjustment.
- To avoid double counting, it was assumed that the disutility associated with each comorbidity was already captured in the BMI-related health state utilities.
- SAEs were assumed to incur disutility in the model. SAE-related disutilities were assumed to be the same for all surgical interventions, namely; ESG, LSG and IGB. This assumption was tested independently for each intervention in the sensitivity analyses.
- In addition, it was assumed that each intervention incurred treatment-related disutility for one cycle in the model, aside from semaglutide which occurred throughout the duration of treatment.
- The disutility of LSG was obtained from a previous study[51] as -0.184. Based on expert clinical advice that the surgical effect of ESG is 25% less severe than that of LSG, the disutility associated with ESG was assumed to be slightly less at -0.138.

Costs:

- Medical problem costs: Given the chronicity of the medical problems, patients were assumed to incur literature derived annual morbidity-specific treatment costs.
- Adverse event costs: Given the acuity of the SAEs, patients with SAEs were assumed to incur literature derived one-off disease treatment costs per adverse event.

Mortality:

- Background mortality was modeled using BMI-specific all-cause mortality hazard ratios applied across health states. These hazard ratios were intended to capture the increased mortality risk associated with obesity and obesity-related disease burden.
- Procedure-related mortality was modeled separately to reflect intervention-specific procedural risk. Procedure-related mortality was assumed to be negligible for ESG, LSG and semaglutide, based on available evidence. For IGB, a procedure-related mortality rate of 0.1% was applied, reflecting published safety data.
- Disease-specific mortality was not modeled separately, as it was assumed to be captured within the BMI-related all-cause mortality estimates.

This table below summarises the key model input parameters, distributions, and sources used in the analyses.

**Table S4: Model parameters and distributions**

| Parameter | Mean % | SE | Distribution | Source |  |
| --- | --- | --- | --- | --- | --- |
| Baseline Characteristics | **Mean** | **SE** |  |  |  |
| Age at the time of intervention | 44 | N/a | N/a | Albon, 2025[4] |  |
| % of females | 87 |  |  | Beran, 2022[5] |  |
| % of males | 13 |  |  | Beran, 2022[5] |  |
| Time horizon | 5 years/Lifetime | N/a | N/a | Albon, 2025[4]/Clinical advice |  |
| Total number of cycles | 6 | N/a | N/a | Clinical advice |  |
| Discount rate costs | 3.50% | N/a | N/a | NICE guidance[6] |  |
| Discount rate outcomes | 3.50% | N/a | N/a | NICE guidance[6] |  |
| Baseline distribution (ESG vs. LSG) |  |  |  |  |  |
| % with Obesity III | 0.3854 | N/a | N/a | CPRD[1] |  |
| % with Obesity II | 0.6146 | N/a | N/a | CPRD[1] |  |
| Baseline distribution (ESG vs. semaglutide, IGB vs. semaglutide) |  |  |  |  |  |
| % with Obesity II | 0.2829 | N/a | N/a | CPRD[1] |  |
| % with Obesity I | 0.7069 | N/a | N/a | CPRD[1] |  |
| Prevalence of medical problems (rate) | | | | | |
| CHD (Unstable Angina/Myocardial infarction): Severe Obesity | 0.023 | 0.001 | Beta (390.777, 16599.527) | Haase *et al*, 2020[7] |  |
| CHD: Obesity 2 | 0.032 | 0.002 | Beta (387.168, 11711.832) |  |  |
| CHD: Obesity 1 | 0.040 | 0.002 | Beta (383.960, 9215.040) |  |  |
| CHD: No obesity | 0.034 | 0.002 | Beta (386.194, 10831.327) |  |  |
| Type 2 diabetes: Severe Obesity | 0.139 | 0.007 | Beta (344.261, 2132.437) |  |  |
| Type 2 diabetes: Obesity 2 | 0.099 | 0.005 | Beta (360.301, 3279.103) |  |  |
| Type 2 diabetes: Obesity 1 | 0.080 | 0.004 | Beta (367.920, 4231.080) |  |  |
| Type 2 diabetes: No obesity | 0.038 | 0.002 | Beta (384.635, 9653.680) |  |  |
| Sleep apneoa: Severe Obesity | 0.018 | 0.001 | Beta (392.782, 21428.440) |  |  |
| Sleep apneoa: Obesity 2 | 0.010 | 0.001 | Beta (395.990, 39203.010) |  |  |
| Sleep apneoa: Obesity 1 | 0.005 | 0.000 | Beta (397.995, 79201.005) |  |  |
| Sleep apnoea: No obesity | 0.005 | 0.000 | Beta (397.995, 79201.005) |  |  |
| CVD (Stroke/TIA): Severe Obesity | 0.018 | 0.001 | Beta (392.782, 21428.440) |  |  |
| CVD (Stroke/TIA): Obesity 2 | 0.020 | 0.001 | Beta (391.980, 19207.020) |  |  |
| CVD (Stroke/TIA): Obesity 1 | 0.026 | 0.001 | Beta (389.574, 14594.041) |  |  |
| CVD (Stroke/TIA): No obesity | 0.023 | 0.001 | Beta (390.588, 16249.830) |  |  |
| Osteoarthritis: Severe Obesity | 0.141 | 0.007 | Beta (343.459, 2092.420) |  |  |
| Osteoarthritis: Obesity 2 | 0.131 | 0.007 | Beta (347.469, 2304.966) |  |  |
| Osteoarthritis: Obesity 1 | 0.130 | 0.007 | Beta (347.870, 2328.053) |  |  |
| Osteoarthritis: No obesity | 0.090 | 0.005 | Beta (363.796, 3665.699) |  |  |
| Mortality Risk | | | | | |
| BMI-specific Mortality Hazard ratios | | | | | |
| Severe obesity | 1.880 | 0.030 | Lognormal (0.631, 0.016) | Bhaskaran,*et al* 2018[8] |  |
| Obesity 2 | 1.360 | 0.010 | Lognormal (0.307, 0.007) |  |  |
| Obesity 1 | 1.120 | 0.010 | Lognormal (0.113, 0.009) |  |  |
| Utilities | | | | | |
| BMI Specific Utilities | | | | | |
| Severe obesity | 0.610 | 0.040 | Beta (90.089, 57.598) | Kelly *et al*, 2023[9] |  |
| Obesity 2 | 0.700 | 0.030 | Beta (162.633, 69.70) |  |  |
| Obesity 1 | 0.780 | 0.030 | Beta (147.940, 41.727) |  |  |
| No obesity | 0.827 | 0.020 | Beta (295.271, 61.897) |  |  |
| Intervention disutilities | | | | | |
| ESG | 0.138 | 0.028 | Beta (21.412, 133.747) | Based on the assumption that the surgical impact of ESG is 25% less severe than LSG |  |
| LSG | 0.184 | 0.037 | Beta (20.216, 89.654) | Sandhu et al, 2023, NICE 2023 [3,10] |  |
| Semaglutide | 0.037 | 0.013 | Beta (7.801, 203.033) | Abramson,*et al* 2019[11] |  |
| IGB | 0.138 | 0.028 | Beta (21.412, 133.747) | Assumed same as ESG |  |
| Adverse events disutilities | | | | | |
| ESG | 0.140 | 0.028 | Beta (21.360, 131.211) | Kelly *et al*,2023[9] |  |
| LSG | 0.140 | 0.028 | Beta (21.360, 131.211) | Assumed same as ESG |  |
| IGB | 0.140 | 0.028 | Beta (21.360, 131.211) | Assumed same as ESG |  |
| Semaglutide | 0.050 | 0.010 | Beta (23.700, 450.300) | NICE, 2023[3] |  |
| Serious adverse events incidence | | | | | |
| ESG | 0.020 | 0.004 | Beta (24.480, 1199.520) | Docimo, *et al* 2023 (pooled estimate)[12] |  |
| LSG | 0.040 | 0.008 | Beta (23.960, 575.040) | Beran *et al* (2022)[5] |  |
| Semaglutide | 0.049 | 0.010 | Beta (23.726, 460.478) | NICE (2023)[3] |  |
| IGB | 0.006 | 0.001 | Beta (24.844, 4115.823) | Albon, 2025[4] & clinical advice |  |
| IGB- Mortality | 0.001 | 0.0002 | Beta (24.974, 24949.026) | Singh, 2020[13] |  |
| Costs | | | | | |
| Intervention costs | | | | | |
| ESG | | | | | |
| ESG procedure | 4409 | 440.9 | Normal (4409, 440.9) | Clinical advice |  |
| Total cost baseline-Y1 | 98.860 | 10 | Normal (98.860, 10) | Clinical advice |  |
| Total cost Y1-Y2 | 45.240 | 4.524 | Normal (45.240,4.524) | Clinical advice |  |
| LSG | | | | | |
| LSG surgery | 6963 | 696.3 | Normal (6963,696.3) | NHS Cost Schedule 2021/22 |  |
| Total cost baseline-Y1 | 98.860 | 10 | Normal (98.860, 10) | Clinical advice |  |
| Total cost Y1-Y2 | 45.240 | 4.524 | Normal (45.240,4.524) | Clinical advice |  |
| Semaglutide | | | | | |
| Cost of Semaglutide Year 1 | 1926.480 | 192.648 | Normal (1926.480, 192.648) | NICE 2023[3] |  |
| Cost of semaglutide Year 2 | 2285.4 | 228.540 | Normal (2285.4, 228.540) | NICE, 2023[3] |  |
| IGB |  |  |  |  |  |
| IGB procedure | 4500 | 450 | Normal (4500,450) | Clinical advice |  |
| Follow-up costY1- Y2 (4 visits to Dietician) | 45.240 | 4.524 | Normal (45.240,4.524) | Clinical advice |  |
| Annual disease management costs | | | | | |
| T2DM | 2290 | 458 | Gamma (25,91.6) | Wang *et al*, 2022[14] |  |
| CHD (MI and Unstable angina) | 1827.531 | 365.506 | Gamma (25,73.101) | NHS cost 2021/22 |  |
| stroke/TIA | 4094.229 | 818.846 | Gamma (25,163.769) | NHS cost 2021/22 |  |
| sleep apnoea | 717 | 143.4 | Gamma (25,28.68) | NHS cost 2021/22 |  |
| osteaoarthritis | 1006.631 | 201.326 | Gamma (25,40.265) | Galvain *et al*, 2021[15] |  |
| Adverse event cost | | | | | |
| ESG | 1403 | 280.520 | Gamma (25.014,56.088) | NHS cost schedule 2021/22 |  |
| LSG | 1403 | 280.520 | Gamma (25.014,56.088) | Assumed same as ESG |  |
| Semaglutide | 154.888 | 30.978 | Gamma (25,6.196) | NICE, 2023[3] |  |
| IGB | 1403 | 280.6 | Gamma (25,56.120) | Assumed same as ESG |  |

**Appendix E: Sensitivity analysis**

This section presents the deterministic and probabilistic sensitivity analyses conducted to explore uncertainty in the model parameters.

Deterministic sensitivity analyses were conducted to evaluate the impact of uncertainty in key model parameters.

Parameters varied included:

- intervention costs
- weight regain assumptions
- treatment effectiveness
- utility values
- adverse event rates

**Table S5: Summary of results from deterministic sensitivity analysis based on ICER (cost per QALY)**

|  | ICER of endoscopic therapy and comparators (£/QALY) | | | | | |
| --- | --- | --- | --- | --- | --- | --- |
|  | **ESG versus LSG** | | **ESG versus Semaglutide** | | **IGB versus Semaglutide** | |
| Base case ICER | £10593.28 | | £7267.07 | | Semaglutide dominant | |
| Sensitivity analysis | Lower value | Upper value | Lower value | Upper value | Lower value | Upper value |
| 1. Varying SAE incidence rates from base-case- values selected based on evidence and clinical advice | | | | | | |
| ESG- SAE rate | £10626.00 | £10256.30 | £6663.86 | £9173.88 | n/a | n/a |
| LSG- SAE rate | £10,347.91 | £11492.00 | n/a | n/a | n/a | n/a |
| IGB- SAE rate | n/a | n/a | n/a | n/a | Semaglutide dominant | Semaglutide dominant |
| Semaglutide- SAE rate | n/a | n/a | £7294.49 | £7233.58 | Semaglutide dominant | Semaglutide dominant |
| 2a. Increasing/decreasing surgery disutilities by 20% | | | | | | |
| ESG disutility | £10285.38 | £10921.09 | £8326.05 | £6447.08 | n/a | n/a |
| LSG disutility | £11037.25 | £10184.43 | n/a | n/a | n/a | n/a |
| IGB disutility | n/a | n/a | n/a | n/a | Semaglutide dominant | Semaglutide dominant |
| *2b. Increasing/decreasing surgery disutilities by 10%* | | | | | | |
| ESG disutility | £10435.00 | £10757.28 | £7768.25 | £6826.65 | n/a | n/a |
| LSG disutility | £10809.73 | £10390.57 | n/a | n/a | n/a | n/a |
| IGB disutility | n/a | n/a | n/a | n/a | Semaglutide dominant | Semaglutide dominant |
| 3. Applying a higher Semaglutide disutility based on evidence | | | | | | |
|  | n/a | n/a | £4927.29 | n/a | Semaglutide dominant | n/a |
| 4a. Increasing/decreasing SAE disutilities by 20% | | | | | | |
| ESG- SAE disutility | £10587.27 | £10600.15 | £7285.88 | £7248.37 | n/a | n/a |
| LSG- SAE disutility | £10606.61 | £10580.84 | n/a | n/a | n/a | n/a |
| IGB- SAE disutility | n/a | n/a | n/a | n/a | Semaglutide dominant | Semaglutide dominant |
| Semaglutide- SAE disutility | n/a | n/a | £7265.20 | £7268.95 | Semaglutide dominant | Semaglutide dominant |
| 4b. Increasing/decreasing SAE disutilities by 10% | | | | | | |
| ESG- SAE disutility | £10590.49 | £10596.93 | £7276.46 | £7257.71 | n/a | n/a |
| LSG- SAE disutility | £10600.15 | £10587.27 | n/a | n/a | n/a | n/a |
| IGB- SAE disutility | n/a | n/a | n/a | n/a | Semaglutide dominant | Semaglutide dominant |
| Semaglutide- SAE disutility | n/a | n/a | £7266.14 | £7268.01 | Semaglutide dominant | Semaglutide dominant |
| 5a. Increasing/decreasing SAE costs by 20% | | | | | | |
| ESG- SAE costs | £10618.00 | £10569.00 | £7163.63 | £7370.52 | n/a | n/a |
| LSG- SAE costs | £10545.00 | £10642.00 | n/a | n/a | n/a | n/a |
| IGB- SAE costs | n/a | n/a | n/a | n/a | Semaglutide dominant | Semaglutide dominant |
| Semaglutide- SAE costs | n/a | n/a | £7294.97 | £7238.97 | Semaglutide dominant | Semaglutide dominant |
| 5b. Increasing/decreasing SAE costs by 10% | | | | | | |
| ESG- SAE costs | £10606.00 | £10582.00 | £7215.35 | £7318.80 | n/a | n/a |
| LSG- SAE costs | £10569.00 | £10618.00 | n/a | n/a | n/a | n/a |
| IGB- SAE costs | n/a | n/a | n/a | n/a | Semaglutide dominant | Semaglutide dominant |
| Semaglutide- SAE costs | n/a | n/a | £7280.97 | £7252.97 | Semaglutide dominant | Semaglutide dominant |
| 6. Aligning CHD prevalence rate with disease severity to account for mortality bias in the literature | | | | | | |
|  | £10,530.20 | | £7278.50 | | Semaglutide dominant | |
| 7a. Increasing/decreasing intervention costs by 20% | | | | | | |
| ESG cost | £14363.70 | £6701.80 | ESG dominant | £23521.31 | n/a | n/a |
| LSG cost | £4482.70 | £16582.86 | n/a | n/a | n/a | n/a |
| IGB cost | n/a | n/a | n/a | n/a | £6030.39 | Semaglutide dominant |
| Semaglutide cost | n/a | n/a | £14376.32 | £175.52 | Semaglutide dominant | Semaglutide dominant |
| 7b. Increasing/decreasing intervention costs by 10% | | | | | | |
| ESG cost | £12448.20 | £8617.30 | ESG dominant | £15394.19 | n/a | n/a |
| LSG cost | £7507.70 | £13557.80 | n/a | n/a | n/a | n/a |
| IGB cost | n/a | n/a | n/a | n/a | Semaglutide dominant | Semaglutide dominant |
| Semaglutide cost | n/a | n/a | £10826.121 | £3725.72 | Semaglutide dominant | Semaglutide dominant |
| 8. Varying the disease severity in the baseline population (lower value-more severe, upper value-less severe) | | | | | | |
|  | £8,045.20 | £13,169 | £11972.20 | £6236.00 | Semaglutide dominant | Semaglutide dominant |
| 9a. Increasing/decreasing health state utilities by 20% | | | | | | |
|  | £13708.40 | £8,701.9 | £7413.71 | £7196.04 | Semaglutide dominant | Semaglutide dominant |
| 9b. Increasing/decreasing health state utilities by 10% | | | | | | |
|  | £11774.40 | £9530.90 | £7411.52 | £7198.11 | Semaglutide dominant | Semaglutide dominant |
| 10a. Increasing/decreasing comorbidity costs by 20% | | | | | | |
|  | £10718.50 | £10468.90 | £7361.60 | £7172.55 | Semaglutide dominant | Semaglutide dominant |
| 10b. Increasing/decreasing comorbidity costs by 10% | | | | | | |
|  | £10656.10 | £10531.00 | £7314.34 | £7219.81 | Semaglutide dominant | Semaglutide dominant |
| 11. Varying long-term transition probabilities based on optimistic (upper value) and pessimistic (lower value) scenarios informed by clinical advice | | | | | | |
| ESG transition probabilities | £8484.80 | £11499.6 | £7849.50 | £4641.32 | n/a | n/a |
| LSG transition probabilities | £28762.16 | £9628.51 | n/a | n/a | n/a | n/a |
| IGB transition probabilities | n/a | n/a | n/a | n/a | Semaglutide dominant | Semaglutide dominant |
| Semaglutide transition probabilities | n/a | n/a | £3679.28 | £26299.03 | Semaglutide dominant | Semaglutide dominant |

*ICER; Incremental cost-effectiveness ratio, ESG; Endoscopic sleeve gastroplasty, LSG; Laparoscopic sleeve gastrectomy, IGB; Intragastric balloon*

**Probabilistic sensitivity analysis**

Probabilistic sensitivity analysis was conducted using Monte Carlo simulation, with parameter distributions assigned according to available evidence. Cost-effectiveness acceptability curves were generated to estimate the probability that each intervention would be cost-effective across willingness-to-pay thresholds. For model 1, the PSA revealed that at a WTP threshold of £20,000, LSG had a 99% probability of being cost-effective when compared to ESG (Figure S1). As shown by the cost-effectiveness plane, the majority of points resided in the northeast (NE) quadrant with sizeable QALY gains at a modest extra cost, indicating that LSG is cost-effective when compared to ESG.

**Figure S1: Cost-effectiveness acceptability curve (left) and cost-effectiveness plane (right) for Model 1**

For model 2, the CEAC (Figure S2) demonstrated that ESG had an 85% probability of being cost-effective when compared to semaglutide at a £20,000 WTP threshold, increasing to 94% at £30,000 WTP threshold. The cost-effectiveness plane demonstrated that ESG is cost-effective since the majority of points are concentrated in the east quadrants, showing increased effectiveness, with either a cost saving or a small additional cost.

**Figure S2. Cost-effectiveness acceptability curve (left) and cost-effectiveness plane (right) for Model 2**

In Model 3, the PSA results show that semaglutide is likely to be cost-effective when compared to IGB at all WTP thresholds (Figure S3). The cost-effectiveness plane shows that most iterations are in the cost saving southern quadrants clustering around the south-eastern quadrant reflecting improved effectiveness.

**Figure S3. Cost-effectiveness acceptability curve (left) and cost-effectiveness plane (right) for Model 3**

**Scenario analysis**

Following clinical advice, the scenario of administering semaglutide for five years, instead of two, was explored (Table S6). In model 2, extending semaglutide treatment to five years led to ESG becoming the dominant option, and for model 3, semaglutide changed from being the dominant option to not being cost-effective when compared to IGB (ICER: £100,147/QALY). These changes in cost-effectiveness were driven by the increased treatment costs from year three to five (+£2,285 per year), and while patients do experience a QALY improvement from sustaining the weight loss up to five years, this is offset by a considerable 0.037 annualised disutility related to the weekly injections.

**Table S6 Scenario analysis for patient taking semaglutide for 5 years**

| **Analysis** | **Cost (£)** | **QALY** | **ICER (£/QALY)** |
| --- | --- | --- | --- |
| **Model 2** (ESG vs 5-year semaglutide)^a^ | | | |
| ESG | 6223.58 | 3.5420 |  |
| Semaglutide (5-year) | 11,930.44 | 3.4934 |  |
| Difference | 5706.89 | 0.0486 | ESG dominant |
| **Model 3** (IGB vs. 5-year semaglutide)^b^ | | | |
| IGB | 6434.32 | 3.4385 |  |
| Semaglutide (5-year) | 11,930.44 | 3.4934 |  |
| Difference |  |  | Semaglutide £100,147/QALY |
| ^a^ Model 2 base-case ESG £7267/QALY (+0.23/£2438)  ^b^ Model 3 base-case semaglutide dominant (-0.049/£603 | | | |

**Appendix F. Model validation**

Model validation was undertaken in line with good practice guidance for economic modeling.

**Internal validation:** Model structure, transition pathways, and calculations were reviewed to ensure logical consistency and correct implementation. Outputs were checked across model cycles to confirm stability and expected behaviour under different parameter inputs.

**External (face) validation:** Model outputs were compared with published evidence and reviewed by the multidisciplinary research team and clinical experts involved in the NIHR-funded program to assess consistency with expected clinical outcomes.

**Health state occupancy checks:** Model-predicted health state occupancy over time was examined for each treatment across the models. The distribution of patients across BMI categories at baseline and at five years, as well as the relative differences between treatment arms, were assessed for clinical plausibility. More effective interventions were expected to result in a higher proportion of patients transitioning to and remaining in lower BMI categories over time. The observed patterns were consistent with expected treatment effects and clinical expectations.

Overall, model outputs were considered consistent with expected clinical and economic patterns for obesity interventions.

Figures S4 to S6 illustrate health state occupancy by treatment arm over time.

**Figure S4: Model 1, ESG versus LSG**

**Figure S5: Model 2, ESG versus Semaglutide**

**Model 3, IGB versus Semaglutide**

**References**

1. UK Health Data Research Alliance. Clinical Practice Research Datalink (CPRD) [Internet]. CPRD database. 2026 [cited 2026 Mar 10]. https://ukhealthdata.org/members/clinical-practice-research-datalink-cprd/. Accessed 10 Mar 2026

2. National Institute for Health and Care Excellence (NICE). NICE HealthTech Guidance HTG711: Endoscopic sleeve gastroplasty for obesity. HealthTech guidance HTG711. 2024.

3. National Institute for Health and Care Excellence (NICE). NICE Overweight and obesity management: Medicines-and-surgery [Internet]. NICE guideline NG246. 2026 [cited 2026 Mar 10]. https://www.nice.org.uk/guidance/ng246/chapter/Medicines-and-surgery. Accessed 10 Mar 2026

4. Albon E, Afentou N, Dretzke J, Hall J, Ogwulu CO, Price MJ, et al. Cost-effectiveness of endoscopic treatments for obesity: a clinical evidence map and systematic review to inform a model-based cost-effectiveness analysis. Health Technol Assess (Rockv). 2025;1–196. https://doi.org/10.3310/PWKQ2310

5. Beran A, Matar R, Jaruvongvanich V, Rapaka BB, Alalwan A, Portela R, et al. Comparative Effectiveness and Safety Between Endoscopic Sleeve Gastroplasty and Laparoscopic Sleeve Gastrectomy: a Meta-analysis of 6775 Individuals with Obesity. Obes Surg. 2022;32:3504–12. https://doi.org/10.1007/s11695-022-06254-y

6. National Institute for Health and Care Excellence (NICE). Guide to the methods of technology appraisal [Internet]. London; 2013 Apr. https://www.nice.org.uk/process/pmg9/resources/guide-to-the-methods-of-technology-appraisal-2013-pdf-2007975843781. Accessed 10 Mar 2026

7. Haase CL, Eriksen KT, Lopes S, Satylganova A, Schnecke V, McEwan P. Body mass index and risk of obesity‐related conditions in a cohort of 2.9 million people: Evidence from a UK primary care database. Obes Sci Pract. 2021;7:137–47. https://doi.org/10.1002/osp4.474

8. Bhaskaran K, dos-Santos-Silva I, Leon DA, Douglas IJ, Smeeth L. Association of BMI with overall and cause-specific mortality: a population-based cohort study of 3·6 million adults in the UK. Lancet Diabetes Endocrinol. 2018;6:944–53. https://doi.org/10.1016/S2213-8587(18)30288-2

9. Kelly J, Menon V, O’Neill F, Elliot L, Combe E, Drinkwater W, et al. UK cost-effectiveness analysis of endoscopic sleeve gastroplasty versus lifestyle modification alone for adults with class II obesity. Int J Obes. 2023;47:1161–70. https://doi.org/10.1038/s41366-023-01374-6

10. Sandhu H, Xu W, Olivieri A-V, Lübker C, Smith I, Antavalis V. Once-Weekly Subcutaneous Semaglutide 2.4 mg Injection is Cost-Effective for Weight Management in the United Kingdom. Adv Ther. 2023;40:1282–91. https://doi.org/10.1007/s12325-022-02423-8

11. Abramson A, Halperin F, Kim J, Traverso G. Quantifying the Value of Orally Delivered Biologic Therapies: A Cost-Effectiveness Analysis of Oral Semaglutide. J Pharm Sci. 2019;108:3138–45. https://doi.org/10.1016/j.xphs.2019.04.022

12. Docimo S, Aylward L, Albaugh VL, Afaneh C, El Djouzi S, Ali M, et al. Endoscopic sleeve gastroplasty and its role in the treatment of obesity: a systematic review. Surgery for Obesity and Related Diseases. 2023;19:1205–18. https://doi.org/10.1016/j.soard.2023.08.020

13. Singh S, de Moura DTH, Khan A, Bilal M, Chowdhry M, Ryan MB, et al. Intragastric Balloon Versus Endoscopic Sleeve Gastroplasty for the Treatment of Obesity: a Systematic Review and Meta-analysis. Obes Surg. 2020;30:3010–29. https://doi.org/10.1007/s11695-020-04644-8

14. Wang H-I, Han L, Jacobs R, Doran T, Holt RIG, Prady SL, et al. Healthcare resource use and costs for people with type 2 diabetes mellitus with and without severe mental illness in England: longitudinal matched-cohort study using the Clinical Practice Research Datalink. The British Journal of Psychiatry. 2022;221:402–9. https://doi.org/10.1192/bjp.2021.131

15. Galvain T, Patel S, Kabiri M, Tien S, Casali G, Pournaras DJ. Cost-effectiveness of bariatric and metabolic surgery, and implications of COVID-19 in the United Kingdom. Surgery for Obesity and Related Diseases. 2021;17:1897–904. https://doi.org/10.1016/j.soard.2021.07.009
